# Rare and common genetic determinants of mitochondrial function determine severity but not risk of amyotrophic lateral sclerosis

**DOI:** 10.1016/j.heliyon.2024.e24975

**Published:** 2024-01-24

**Authors:** Calum Harvey, Marcel Weinreich, James A.K. Lee, Allan C. Shaw, Laura Ferraiuolo, Heather Mortiboys, Sai Zhang, Paul J. Hop, Ramona A.J. Zwamborn, Kristel van Eijk, Thomas H. Julian, Tobias Moll, Alfredo Iacoangeli, Ahmad Al Khleifat, John P. Quinn, Abigail L. Pfaff, Sulev Kõks, Joanna Poulton, Stephanie L. Battle, Dan E. Arking, Michael P. Snyder, Project MinE ALS Sequencing Consortium, Jan H. Veldink, Kevin P. Kenna, Pamela J. Shaw, Johnathan Cooper-Knock

**Affiliations:** a Sheffield Institute for Translational Neuroscience (SITraN), University of Sheffield, Sheffield, UK; b Clinical Neurobiology, German Cancer Research Center and University Hospital Heidelberg, Germany; c Department of Epidemiology, University of Florida, Gainesville, FL, USA; d Department of Neurology, Brain Center Rudolf Magnus, University Medical Center Utrecht, Utrecht, the Netherlands; e Division of Evolution, Infection and Genomics, School of Biological Sciences, The University of Manchester, Manchester, UK; f King's College London, Institute of Psychiatry, Psychology and Neuroscience, Department of Basic and Clinical Neuroscience, London, UK; g Department of Pharmacology and Therapeutics, Institute of Systems, Molecular & Integrative Biology, Liverpool, UK; h Perron Institute for Neurological and Translational Science, Perth, Australia; i Centre for Molecular Medicine and Innovative Therapeutics, Murdoch University, Perth, Australia; j Nuffield Department of Obstetrics and Gynaecology, University of Oxford, Oxford, UK; k McKusick-Nathans Institute, Department of Genetic Medicine, Johns Hopkins University School of Medicine, Baltimore, MD, USA; l Center for Genomics and Personalized Medicine, Stanford University School of Medicine, Stanford, CA, USA

## Abstract

Amyotrophic lateral sclerosis (ALS) is a fatal neurodegenerative disease involving selective vulnerability of energy-intensive motor neurons (MNs). It has been unclear whether mitochondrial function is an upstream driver or a downstream modifier of neurotoxicity. We separated upstream genetic determinants of mitochondrial function, including genetic variation within the mitochondrial genome or autosomes; from downstream changeable factors including mitochondrial DNA copy number (mtCN). Across three cohorts including 6,437 ALS patients, we discovered that a set of mitochondrial haplotypes, chosen because they are linked to measurements of mitochondrial function, are a determinant of ALS survival following disease onset, but do not modify ALS risk. One particular haplotype appeared to be neuroprotective and was significantly over-represented in two cohorts of long-surviving ALS patients. Causal inference for mitochondrial function was achievable using mitochondrial haplotypes, but not autosomal SNPs in traditional Mendelian randomization (MR). Furthermore, rare loss-of-function genetic variants within, and reduced MN expression of, ACADM and DNA2 lead to ∼50 % shorter ALS survival; both proteins are implicated in mitochondrial function. Both mtCN and cellular vulnerability are linked to DNA2 function in ALS patient-derived neurons. Finally, MtCN responds dynamically to the onset of ALS independently of mitochondrial haplotype, and is correlated with disease severity. We conclude that, based on the genetic measures we have employed, mitochondrial function is a therapeutic target for amelioration of disease severity but not prevention of ALS.

## 1 Introduction

Amyotrophic lateral sclerosis (ALS) is a rapidly progressive, relatively common and incurable neurodegenerative disease. In the majority of cases, ALS is thought to result from a complex interaction of genetic and environmental risk factors [1]. The onset of ALS is distinct from its progression. Whilst disease can take >50 years to develop, paralysis as a result of motor neuron (MN) loss often occurs within <5 years from symptom onset [2]. Specific genetic [3,4] and environmental factors [5] have been associated with severity of the clinical phenotype after development of disease.

Inadequate mitochondrial energy production has long been associated with neurodegenerative disease [[6], [7], [8]], but questions have remained regarding the position of energy deficit in the pathophysiological cascade [9]. It has been difficult to establish whether energy deficit is an upstream determinant of disease, a downstream consequence of neurodegeneration, or an adjunct which accelerates neuronal death caused by other factors. Primary mitochondrial disease is associated with sensorimotor neuropathy, but specific toxicity to motor neurons is not a cardinal feature [10]. However, several lines of evidence exist linking ALS pathology and mitochondrial dysfunction: TDP-43 mislocalization, which is the hallmark of >97 % of ALS cases [11], is associated with TDP-43 entry into the mitochondria and impaired bioenergetic function [12]. Moreover, in limbic-predominant age-related TDP-43 encephalopathy (LATE), TDP-43 pathology has been associated with reduced mitochondrial DNA copy number (mtCN) in cortical tissue [13]. Treatments for ALS aimed at reducing a bioenergetic deficit have so far proved ineffective [14].

One difficulty in studying mitochondrial energy production is the method employed for measurement of bioenergetic function. MtCN is a measure of the number of mitochondria within a biological sample. Higher copy number corresponds to a greater number of mitochondria and potentially a greater capacity for energy production [15], although this relationship is not linear [16]. MtCN varies throughout life and adapts to the particular metabolic needs of each specific cell and cell-type [17]. MtCN can also be modified by environmental factors including the onset of disease and ageing [18], which makes it difficult to differentiate the effect of mtCN on disease from the effect of disease on mtCN. In contrast to mtCN a significant proportion of mitochondrial DNA sequence is fixed at conception; as a result mitochondrial haplotypes are necessarily upstream of a late age of onset disease such as ALS. This is distinct from mitochondrial DNA mutations accrued through life which are detectable as mitochondrial heteroplasmy.

We took advantage of a specific set of mitochondrial haplotypes which have been robustly associated with functional readouts including mtCN, mtCN-associated quantitative traits, and non-cancer mortality [19]. Our objective was to use mitochondrial haplotypes as a measure of upstream variation in mitochondrial function independent of the effect of disease onset. We tested for an effect of mitochondrial haplotype on ALS risk and/or survival. In addition, a number of nuclear encoded genes have been associated with mitochondrial function [20] and genetic variation within nuclear encoded genes is also largely fixed at conception; therefore, we performed a gene burden analysis as an orthogonal test of a causal link between mitochondrial function and ALS risk and/or survival. The mitochondrial haplotypes we studied are common and likely represent physiological variation of mitochondrial function with small effect sizes. In contrast our gene burden analysis focused on rare genetic variants with large effect sizes, which may be viable therapeutic targets.

Our approach is distinct from previous studies of mitochondrial haplogroups in the context of ALS [21,22]. Haplogroups consist of sets of mitochondrial DNA mutations defined by their association with a particular ancestry group. In contrast we utilise haplotypes defined by their functional correlates. Our narrow approach in which we exclude haplotypes without functional evidence, adds power to answer the question as to whether mitochondrial function is truly associated with ALS risk or severity.

In our analysis, genetically-determined mitochondrial function does not affect the risk of developing ALS. However, we do find evidence for modification of ALS survival by mitochondrial function, suggesting that this may be a valid therapeutic target for a majority of ALS patients. DNA2 is a nuclear encoded gene linked to mitochondrial function. In a series of convergent lines of evidence we demonstrate that shorter ALS survival is associated with loss-of-function mutations within DNA2 and with reduced expression of DNA2 within iPSC-derived motor neurons. Moreover, *in vitro* we demonstrated that vulnerability of patient-derived neurons carrying an ALS-associated C9ORF72 expansion [23] to pharmacological inhibition of DNA2, is linked to mtCN. Our findings have important implications for efforts to understand the role of energy deficit in the pathophysiology of ALS and could potentially lead to new treatment approaches.

## 2 Results

### 2.1 Mitochondrial genotype is an upstream determinant of ALS survival but not ALS risk

We took advantage of mitochondrial haplotypes fixed at conception, which have been robustly associated with mtCN, with mtCN-associated quantitative traits, and with disease [19] (**STAR Methods: ‘Mitochondrial haplotype’**). We infer that these mitochondrial haplotypes are associated with mitochondrial function. By dividing ALS and control genomes based on mitochondrial haplotype we were able to explore whether mitochondrial function is an upstream determinant of ALS risk and/or survival. Initially we used WGS of DNA extracted from 5,635 sporadic ALS patients and 2,238 controls [24] from Project MinE (www.projectmine.com, **STAR Methods: ‘Study cohort’**).

**Fig. 1:**
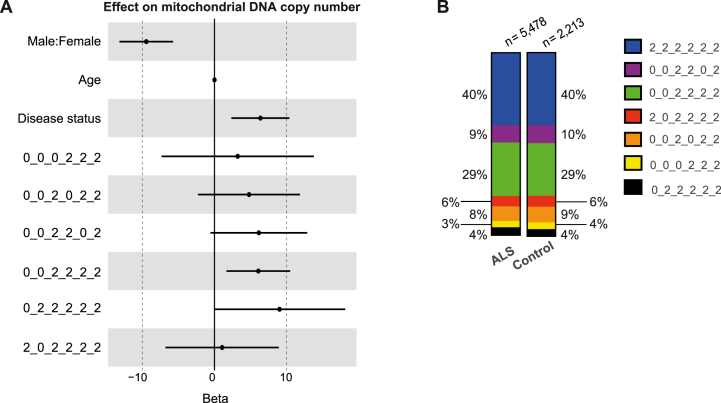
**Mitochondrial haplotype is linked to mitochondrial copy number and ALS survival but not ALS risk.** (A) Forest plot for the effect of mitochondrial haplotype, sex, age and disease status on mitochondrial copy number by multivariable linear regression. (B) Relative proportions of measured mitochondrial haplotypes amongst ALS patients and controls. Mitochondrial haplotypes were determined by six mitochondrial SNPs: MT73A_G, MT7028C_T, MT10238T_C, MT7028C_T, MT12612A_G, MT13617T_C, MT15257G_A. Haplotypes are displayed in the order MT73_MT7028_MT10238_MT12612_MT13617_MT15257, with 2 as the reference allele, 0 as the alternate allele.

First we validated the link between mitochondrial haplotypes and mtCN in our dataset. MtCN is itself a complex phenotype determined by interaction of host genetics and the environment [25]. MtCN is cell-specific [18] and ALS is known to alter the proportions of blood leukocytes [4]. To overcome this limitation we used a subset of our whole blood WGS cohort for whom we could derive proportions of WBC based on DNA methylation [26] (**STAR Methods: ‘Mitochondrial DNA copy number’ and ‘White blood cell proportions’**). This subset included 3,549 ALS patients and 1,529 controls. As expected, specific mitochondrial haplotypes were associated with mtCN in our dataset after adjusting for WBC proportions (0_0_2_2_0_2 p = 7.25e-2, OR = 474; 0_0_2_2_2_2 p = 7.00e-3, OR = 444; 0_2_2_2_2_2, p = 0.05, OR = 8.42e3, multivariable linear regression, Fig. 1A, Supplementary Table 1, **STAR Methods: Statistical analysis including mitochondrial haplotypes and mtCN**). Our findings match a previous study of the same haplotypes in peripheral blood [19].

We did not find any evidence that mitochondrial haplotype was associated with ALS risk (p > 0.05, logistic regression, Fig. 1B). Interestingly, mitochondrial haplotype was significantly associated with ALS survival (p = 0.0096, Chisq = 16.91, ANOVA, Supplementary Fig. 1C, Supplementary Table 2). After correction of left truncation bias mitochondrial haplotype was still significantly associated with ALS survival (p = 0.023, Chisq = 14.7, ANOVA, Supplementary Fig. 3A, Supplementary Table 3). In our analysis we excluded diagnostic delay (time from symptom onset to diagnosis) as a covariate because of some concern that this may be determined in part by mitochondrial haplotype. Interestingly when diagnostic delay is included as a covariate then the effect of mitochondrial haplotype is actually enhanced (p = 1.9e-04, Chisq = 26.3, ANOVA, Supplementary Fig. 3B, Supplementary Table 4) suggesting that mitochondrial haplotype has an outsized effect on survival time after diagnosis. Survival data included 4,549 patients who had died and 930 censored data points where the patient is still alive.

Mitochondrial haplotype is fixed at conception and is therefore necessarily upstream of a late age of onset disease such as ALS. Our data suggest that mitochondrial dysfunction determined at conception is not a risk factor for ALS but mitochondrial function can affect the progression of ALS once disease has been initiated.

#### 2.1.1 Mitochondrial genotype is associated with ALS survival in an independent cohort

To validate our findings, we obtained WGS data from an additional cohort including 843 ALS patients (www.answerals.org, **STAR Methods: ‘Study cohort’**). In this analysis mitochondrial haplotype was still significantly associated with ALS survival (p = 0.047, Chisq = 12.7, ANOVA, Supplementary Fig. 1D, Supplementary Table 5). This validation cohort was enriched with long survivors compared to the test cohort (median survival 3.15 years versus 2.72 years). Given the differences in the survival profile of the two cohorts we hypothesised that the relative proportions of specific mitochondrial haplotypes would also vary between cohorts. Consistent with this idea, the most protective mitochondrial haplotype from the test cohort (0_0_0_2_2_2, Supplementary Fig. 1C) was overrepresented in the validation cohort (Supplementary Fig. 1B). In fact, the association between the 0_0_0_2_2_2 haplotype and longer ALS survival was statistically significant in isolation in the validation cohort (p = 0.023, HR = 0.26; Cox regression, Supplementary Fig. 1D) and remained significant after correction for left truncation bias (p = 0.026, HR = 0.26, Supplementary Fig. 3C, Supplementary Table 6).

We utilised a third smaller cohort of long surviving ALS patients from Turkey and Portugal to further examine the role of the putative protective 0_0_0_2_2_2 mitochondrial haplotype. These individuals formed part of the test cohort but were removed from the initial analysis due to an atypical long survival profile (**STAR Methods: ‘Sample quality control’**, Supplementary Fig. 1A). We wondered whether the protective 0_0_0_2_2_2 mitochondrial haplotype might be over-represented in this group, as it is in the validation cohort. Consistent with our hypothesis, the protective mitochondrial haplotype is overrepresented in these individuals compared to the remainder of the test cohort (p = 4e-3, Chisq = 8.27). This is consistent with a neuroprotective role for the 0_0_0_2_2_2 mitochondrial haplotype.

### 2.2 Mitochondrial copy number is elevated in ALS patient tissues

**Fig. 2:**
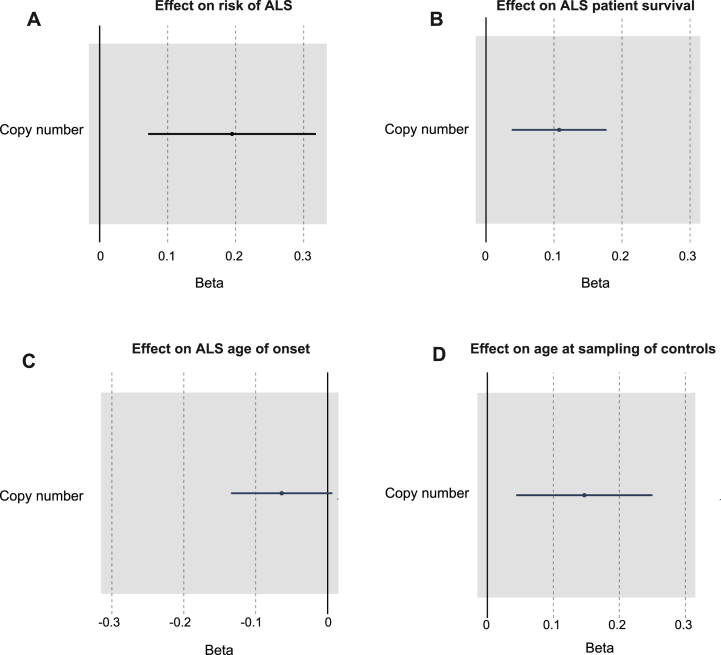
**Mitochondrial copy number is linked to ALS survival and age of disease onset.** (A) Forest plot for the correlation between mitochondrial copy number and ALS risk by multivariable logistic regression. (B) Forest plot for the correlation between mitochondrial copy number and ALS patient survival by Cox regression. (C) Forest plot for the correlation between mitochondrial copy number and ALS age of onset by Cox regression. (D) Forest plot for the correlation between mitochondrial copy number and age at sampling in controls by Cox regression. Beta refers to effect size per 100 unit change in mtCN.

We have suggested that mitochondrial function is not an upstream cause of ALS. We next asked whether the initiation of the ALS disease process impacts upon mtCN. mtCN is a determinant of mitochondrial function but, unlike mitochondrial haplotype, it can vary in response to disease. We tested for a significant relationship between ALS diagnosis and mtCN after adjusting for WBC proportions and mitochondrial haplotype (**STAR Methods: ‘Statistical analysis including mitochondrial haplotypes and mtCN’**). Consistent with a change in mtCN in response to disease, mtCN is significantly elevated in ALS patients compared to controls (p = 1.98e-3, n = 3,549 ALS patients and n = 1,529 controls, multivariable logistic regression, OR = 1.2 per 100 unit change in mtCN, Fig. 2A, Supplementary Table 7).

### 2.3 Mitochondrial copy number is associated with ALS severity

We have shown that mitochondrial function is an upstream modifier of ALS severity. Moreover, our analysis of mtCN suggests that the onset of ALS itself may precipitate an increase in mtCN. We hypothesised that changes in mtCN may be proportional to disease severity independently of the effect of mitochondrial haplotype. For example patients who achieve a higher mtCN may be more likely to avoid neurotoxic bioenergetic stress, or else a more aggressive disease course may precipitate a greater increase in mtCN. To test this hypothesis, we examined whether mtCN was correlated with disease severity within our WGS dataset after correcting for both WBC proportions and mitochondrial haplotype (**STAR Methods: ‘Statistical analysis including mitochondrial haplotypes and mtCN’**). In this analysis higher mtCN is significantly associated with shorter ALS survival (p = 0.0024, n = 3,549 ALS patients and n = 1,529 controls, HR = 1.1 per 100 unit change in mtCN, Cox regression, Fig. 2B–Supplementary Table 8).

### 2.4 The link between mitochondrial copy number and age is modified by ALS

Age is negatively correlated with mtCN measured in peripheral blood [19]. If ALS is a consistent modifier of mtCN throughout the disease course, we questioned whether the normal relationship between mtCN and age might be altered.

Higher mtCN is non-significantly associated with higher age at blood sampling for ALS patients (p = 0.08, n = 3,549, HR = 0.94 per 100 unit change in mtCN, Cox Regression, Fig. 2C–Supplementary Table 9, **STAR Methods: ‘Statistical analysis including mitochondrial haplotypes and mtCN’**). In contrast, in our control cohort the direction of association is reversed (p = 5.3e-03, n = 1,529, HR = 1.1 per 100 unit change in mtCN, Cox regression, Fig. 2D–Supplementary Table 10). This is consistent with previous literature demonstrating lower mtCN with advanced age in neurologically normal individuals [19]. We conclude that the biological mechanism mediating changes in mtCN in response to ALS is dynamic over time and is independent of the effects of normal ageing. Mitochondrial haplotype is not significantly associated with ALS age of onset (p = 0.24, Chisq = 7.9, ANOVA, Supplementary Fig. 3D, Supplementary Table 11).

### 2.5 Mendelian randomization (MR) analysis of mitochondrial DNA copy number

Our use of mitochondrial haplotypes which are associated with mtCN for causal inference is comparable to a MR analysis of genetic liability to higher mtCN. We have previously demonstrated that traditional MR using autosomal SNPs to infer mtCN is prohibited by high instrument pleiotropy and limited power [19]. Nevertheless, others have applied this approach [27] and therefore we used a previous GWAS of mtCN [19] to derive a set of instrumental SNPs for inference of mtCN. Applying this to both ALS risk [4] and ALS severity in the Project MinE dataset (**STAR Methods: ‘Mendelian randomization’**) did not identify a significant association between genetic liability to higher mtCN and ALS risk (p > 0.05, Supplementary Fig. 4A) or severity (p > 0.05, Supplementary Fig. 4B) using the IVW test, or the weighted median, weighted mode or MR Egger methods which are relatively robust to instrument pleiotropy [28] (Supplementary Table 12). Overall, we conclude that a traditional MR analysis using autosomal SNPs is not able to reproduce our result, thereby demonstrating the value of using mitochondrial haplotypes.

### 2.6 Mitochondrial genotype does not have the same effect irrespective of the cause of neurodegeneration

We wondered whether the effect of mitochondrial haplotypes on disease progression was specific to ALS or whether we might discover the same effect for other causes of neurodegeneration. To test this hypothesis we obtained an additional WGS cohort including 262 Parkinson's disease (PD) patients (www.ppmi-info.org, **STAR Methods: ‘Study cohort’**). Survival in PD is multifactorial and associated with a higher degree of missingness compared to ALS; therefore we relied on the rate of change in the UPDRS measured at 5 years after symptom onset [29]. In this analysis mitochondrial haplotype was not associated with rate of disease progression (p = 0.56, sum of squares = 30.0, ANOVA, Supplementary Fig. 5, Supplementary Table 13). The PD dataset contains fewer samples than the ALS dataset and it is possible that the lack of effect was due to a relatively unpowered test. Against this possibility is the fact that direction of effect is not maintained for any individual haplotype compared to the ALS analysis (Supplementary Fig. 1C, Supplementary Fig. 5).

### 2.7 Function of nuclear encoded genes linked to mitochondrial function impacts on ALS survival

**Fig. 3:**
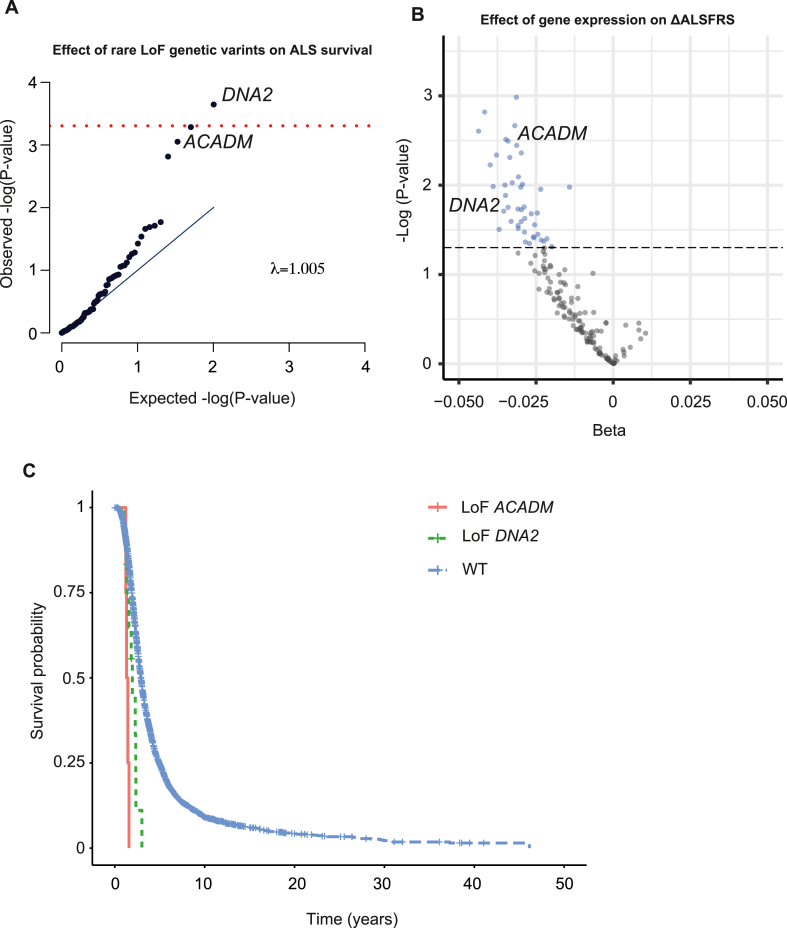
**Motor neuron expression of nuclear encoded genes associated with mitochondrial function is associated with rate of disease progression.** (A) QQ-plot for expected and observed P-values in Cox regression including the effect of burden of rare loss of function (LoF) genetic variants within 166 nuclear encoded genes associated with mitochondrial function, on ALS survival. The test statistics show no evidence of inflation (λ = 1.005). One gene, DNA2, is significant after Bonferroni multiple testing (indicated by red line). (B) Volcano plot including effect sizes and test statistics for multivariable linear regression including the effect of expression of nuclear encoded genes associated with mitochondrial function on rate of ALS progression. Black dotted line and blue circles indicate p < 0.05. (C) Kaplan-Meier curve reveals the reduction in survival for ALS patients carrying LoF variants within *DNA2* and *ACADM* compared to the overall Project MinE cohort. (For interpretation of the references to colour in this figure legend, the reader is referred to the Web version of this article.)

Next we analysed loss-of-function (LoF) genetic variants (**STAR Methods: ‘Rare variant association testing’**) within nuclear encoded genes associated with mitochondrial function to determine whether they might be linked to ALS rate of progression. Compared to mitochondrial haplotypes, LoF variants are typically rare with large effect sizes and therefore we hypothesised that we may discover a putative therapeutic target linked to mitochondrial function. We obtained a published and curated list of 166 nuclear encoded genes associated with mitochondrial function and used to study neurodegenerative disease [20]. Using the Project MinE dataset we tested whether LoF genetic variants within the 166 nuclear encoded genes associated with mitochondrial function [20], could influence ALS survival. Like mitochondrial haplotypes, genetic variation within nuclear encoded genes is fixed at conception and therefore necessarily upstream of a late age of onset disease such as ALS. Compared to the exome overall, the median p-value for mitochondrial genes was significantly lower than expected (p = 0.004, permutation test). One gene, *DNA2*, was significantly enriched with LoF mutations in ALS patients with shorter survival after Bonferroni multiple testing correction (p = 2.3e-4, beta = 1.17, Cox regression, Fig. 3A, Supplementary Table 14); mean survival in twelve ALS patients carrying a LoF mutation in *DNA2* was 1.83 years compared to 3.46 years in patients without a LoF mutation within *DNA2* (Fig. 3C).

To develop this finding in a cell-specific context we analysed MN expression of the same 166 nuclear genes associated with mitochondrial function. We utilised RNA-sequencing from iPSC-derived motor neurons obtained from 180 ALS patients (www.answerals.org). RNA-sequencing data and survival data were available for only 82 individuals because many of the patients were not followed-up until death. Therefore we focused on the link between MN gene expression and rate of change in the ALSFRS-R which was available for all 180 patients. Rate of change in the ALSFRS-R is frequently used to measure ALS progression [30]. We tested for an association between MN expression of nuclear encoded mitochondrial genes with rate of change in the ALSFRS-R by multivariable linear regression (Fig. 3B–Supplementary Table 15, **STAR Methods: ‘Transcriptome analysis’**) but no gene was significant after Bonferroni multiple testing correction. However, compared to the background transcriptome the median p-value for mitochondrial genes was significantly lower than expected (p = 0.0059, permutation test). Moreover, for the 44 genes, including DNA2, which achieved nominal significance (p < 0.05) expression was inversely related to rate of change in the ALSFRS-R (Fig. 3A) meaning that lower expression is linked to increased rate of progression. This was significant compared to the direction of correlation for the background transcriptome (p = 5e-4, Fisher Exact test). We conclude from this analysis that mitochondrial function within MN is associated with the rate of ALS progression; this orthogonal validation is consistent with our earlier analysis of mitochondrial haplotype and mtCN.

To leverage additional statistical power, we performed a meta-analysis of our MN expression and LoF analyses which revealed that two genes were significantly associated with ALS severity after Bonferroni multiple testing correction (*ACADM*: p = 1.76e-05; and *DNA2*: p = 4.44e-05; Stouffer's test). Both genes are still significant after Bonferroni multiple testing correction even if a stringent correction for left truncation bias is applied (*ACADM*: p = 1.46e-04; and *DNA2*: p = 2.40e-04; Stouffer's test). It is notable that for both *ACADM* and *DNA2*, LoF mutations and lower MN gene expression are associated with faster disease progression; moreover both genes are at least nominally significant in both analyses (Supplementary Table 14 and Supplementary Table 15). Mean survival in four ALS patients carrying a LoF mutation in *ACADM* was 1.39 years compared to 3.46 years in patients without a LoF mutation within *ACADM* (Fig. 3C). LoF mutations within *DNA2* or *ACADM* are not associated with risk for ALS (p > 0.5, Firth logistic regression).

### 2.8 Inhibition of DNA2 in ALS patient-derived iNeurons reduces viability in a manner proportional to mtCN

**Fig. 4:**
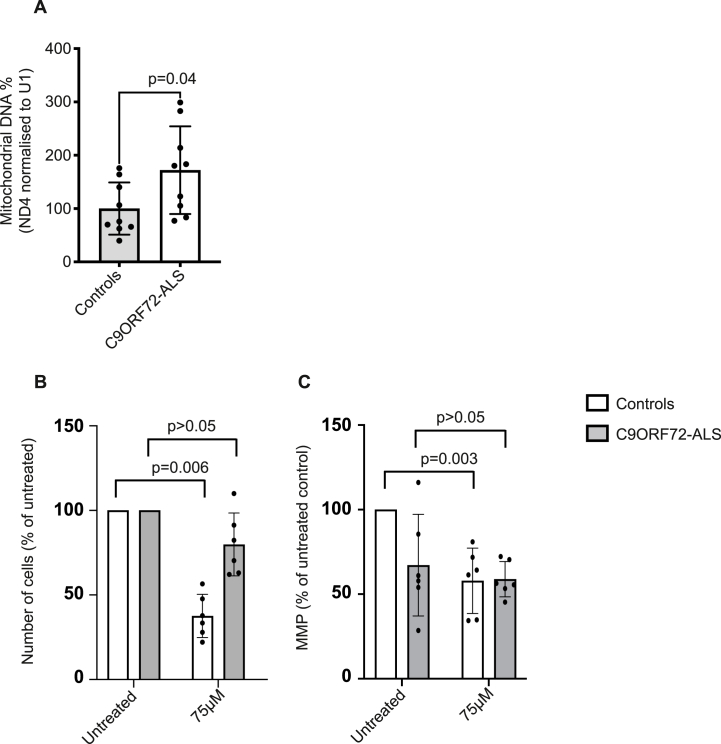
**The effect of DNA2 function on cellular viability is mediated via mitochondrial function in ALS patient-derived iNeurons.** (A) Measurement of mtCN measured by qPCR for mitochondrial gene ND4 normalised to U1. Comparison between iNeurons derived from neurologically normal controls and *C9ORF72*-ALS patients. Three biological replicates were performed per condition and three technical replicates per biological replicate (cell line). (B) Nuclei counts and (C) mitochondrial membrane potential (MMP) before and after treatment with 75 μm of C5 DNA2 inhibitor for iNeurons derived from neurologically normal controls and *C9ORF72*-ALS patients. Two biological replicates were performed per condition and three technical replicates per biological replicate. All p-values shown for Welch's *t*-test; error bars are standard deviation.

We have linked ALS survival to function of DNA2; we hypothesise that this relationship is mediated via an effect on mitochondrial function. To test this hypothesis we modulated DNA2 function within an *in vitro* model of ALS where we could also measure markers of mitochondrial function. iNeurons were differentiated from three ALS patients carrying a pathogenic *C9ORF72* mutation and three unaffected, age and sex matched controls (**STAR Methods: ‘iNPC tissue culture, iNeuron differentiation and DNA2 inhibitor treatment’,** Supplementary Table 16**).** Mirroring our WGS analysis, mtCN was elevated in diseased iNeurons compared to control iNeurons (p = 0.04, Welch's *t*-test, Fig. 4A, **STAR Methods: ‘DNA extraction and mtDNA quantification for iNeurons’**) in keeping with a compensation for disease. Consistent with this idea, when we applied an inhibitor of DNA2, cellular viability and mitochondrial membrane potential (MMP) were reduced in a manner proportional to mtCN. After treatment with 75 μm of C5 DNA2 inhibitor the reduction in the number of surviving cells, as measured by nuclei counts (**STAR Methods: ‘Live cell imaging assays of iNeurons’**) was significantly correlated with baseline mtCN across all individual cell lines (p = 5e-4, rho = −0.85, Spearman correlation); the same was true for MMP (p = 0.035, rho = +0.61, Spearman correlation). As expected, given the difference in baseline mtCN, this led to a significant difference between patient and control derived cells: after treatment with 75 μm of C5 DNA2 inhibitor the number of nuclei was reduced for control iNeurons (FC = 0.33, p = 0.006, Welch's *t*-test, Fig. 4B) but not for *C9ORF72*-ALS iNeurons (FC = 0.89, p = 0.41, Fig. 4B); similarly MMP was reduced for control iNeurons (FC = 0.58, p = 0.003, Welch's *t*-test, Fig. 4C) but not for *C9ORF72*-ALS iNeurons (FC = 0.93, p = 0.28, Fig. 4C). MMP was however reduced in *C9ORF72*-ALS iNeurons under basal conditions (FIg 4C), this is consistent with previous literature [31] and may indicate a reason for the compensatory increase in mtCN observed at baseline.

Overall, we demonstrate a disease-associated compensatory increase in mtCN which is coincident with reduced vulnerability to DNA2 inhibition. This is consistent with our genetic data and supports the idea that enhanced DNA2 function might be a viable therapeutic target for amelioration of ALS survival given that ALS survival is linked to mitochondrial function. Importantly we do not propose DNA2 as a cause of disease, but rather a modifier of neurotoxicity in the face of an insult which impairs mitochondrial function.

## 3 Discussion

ALS is a result of specific toxicity to MN which leads to death, usually from respiratory failure, within 2–5 years [2]. The majority of ALS cases are thought to be the product of a gene-environment interaction [1]. In all cases described to date, genetic risk is present from conception but disease does not develop until much later. The contrast between the decades it takes to develop disease and the relatively few years it takes for disease to spread through the CNS [2] has led to the suggestion that these two processes are biologically distinct. In this study we have treated disease risk and disease progression as separate entities, and have sought to examine the relationship of each to variation in mitochondrial function.

The role of bioenergetics in neurodegenerative disease is controversial and has been the subject of a number of failed clinical trial interventions for ALS, e.g. the phase 3 dexpramipexole trial [14]. Significant evidence exists from both *in vitro* [32] and *in vivo* [33] studies suggesting that insufficient energy production exacerbates or even causes neurotoxicity. Mitochondria are the source of cellular ATP production and, down-regulation of mitochondrial function at disease onset is associated with a more severe phenotype in the *SOD1*-ALS mouse model [34]. What has been missing until now is a study of pre-morbid mitochondrial function across a large number of ALS patients to determine the relative effect on disease risk and severity.

To isolate the upstream role of mitochondrial function we used mitochondrial genetic haplotypes with a validated link to mitochondrial function [19]. Mitochondrial haplotypes are fixed at conception and therefore our analysis is not impacted by secondary effects of disease. Our approach is analogous to Mendelian randomization (MR) but traditional MR using autosomal SNPs linked to mtCN, is prohibited by high instrument pleiotropy and limited power [19]. Indeed, here we have demonstrated the superiority of mitochondrial haplotypes through comparison with traditional MR using the same ALS patient cohort.

We did not observe a significant link between mitochondrial haplotype and risk of developing ALS which suggests that genetically-determined mitochondrial function is not a primary cause of the selective degeneration of motor neurons seen in ALS. This is useful information for the field and suggests that therapeutic intervention to modify mitochondrial function is not appropriate in, for example, asymptomatic family members of ALS patients who harbour disease-causing mutations. We have identified a significant relationship between mitochondrial haplotype and ALS survival which we validated in an independent cohort. Our analyses suggest that interventions to modify mitochondrial function may be able to slow disease progression and increase survival time.

We provide evidence that mtCN in blood changes dynamically through the course of ALS independently of mitochondrial haplotype. MtCN was elevated in the blood of ALS patients and significantly correlated with survival, but interpretation is difficult, particularly because mtCN is tissue specific [17] and ALS is a disease of the CNS. A limitation of our analysis is that our estimate of mtCN failed to include platelet counts because this information was not available. However, despite this, we were able to replicate previously reported results including the association of mtCN with specific mitochondrial haplotypes and with age in control individuals [19].

Our analysis of mitochondrial haplotype and mtCN established an upstream role for physiological variation of mitochondrial function in ALS disease progression, but the design of therapeutic interventions may require targets with larger effect sizes. We achieved this through a further study of genetic variation within nuclear encoded genes associated with mitochondrial function. By performing rare variant association testing using WGS we were able to associate LoF of nuclear encoded mitochondrial genes with reduced ALS survival. For two genes – *DNA2* and *ACADM* – we observed ∼50% reduction in survival time for patients carrying rare LoF mutations. Transcriptome analysis in iPSC-derived MN from ALS patients confirmed that the level of expression of nuclear encoded mitochondrial genes is significantly associated with the rate of ALS progression in the primary cell of interest. A meta-analysis of our rare variant burden testing and transcriptome analysis revealed that the relationship between reduced function of *DNA2* and *ACADM* and shorter ALS survival was significant after Bonferroni multiple testing correction; and *DNA2* was significant in the rare variant analysis in isolation.

*ACA*DM encodes the medium-chain specific (C4 to C12 straight chain) acyl-coenzyme A dehydrogenase. Increased expression of this protein has been identified in the spinal cord of G93A-SOD1-ALS mice [35]. This enzyme is key in the process of fatty acid beta-oxidation for energy production. Importantly, medium-chain fatty acids are able to bypass the carnitine shuttle which is necessary for import of long chain fatty acids into mitochondria; defects in the carnitine shuttle have previously been associated with ALS [36]. A genetic defect leading to deficient metabolism of medium-chain fatty acids might exacerbate this defect by removing a potential escape mechanism.

The second nuclear encoded mitochondrial gene we identified with a large effect on ALS survival was *DNA2* which encodes a DNA replication helicase/nuclease 2 involved in DNA repair, particularly within the mitochondria. Biallelic variants within *DNA2* have been associated with a developmental phenotype [37] but our data suggest that heterozygous LoF changes in this gene may impair the bioenergetic response to ALS leading to accelerated disease progression, perhaps via excessive DNA damage within the mitochondrial genome. Using an *in vitro* model of ALS, we were able to demonstrate a link between DNA2 and readouts of mitochondrial function; the toxic effect of DNA2 inhibition was ameliorated by higher baseline mtCN. This is consistent with our earlier conclusion that ALS survival is linked to mitochondrial function and can be adversely affected by rare LoF mutations within DNA2. We suggest that enhancing DNA2 function may be an effective therapeutic target; even if DNA2 dysfunction is not a disease-associated insult in the majority of ALS cases.

It is possible that mitochondrial dysfunction accrued through life could have an effect on ALS risk that is not captured by our analysis. These effects could even manifest via acquired mutations within mitochondrial DNA leading to mitochondrial heteroplasmy. However, due to difficulty in assigning the direction of causation we have not we have not considered mitochondrial heteroplasmy in this study.

Interestingly we did not observe a similar relationship between mitochondrial haplotype and PD rate of progression. One interpretation is that the relationship we observe is specific to MN health and not a nonspecific feature of neurodegeneration. Of course, mitochondrial dysfunction has been associated with PD [38] and it may be that the physiological variation determined by mitochondrial haplotype is outweighed by a disease-specific process. It should be noted that our analysis of PD contained fewer samples and was relatively underpowered compared to the ALS analysis, so this work will require further validation. Moreover, we are not able to comment on whether mitochondrial function is a modifier of disease progression for other types of neurodegeneration such as Alzheimer's disease.

Taken together our work links bioenergetic function within MN to rate of progression of ALS. In addition, we have shown that upstream genetic determinants of bioenergetic function are not linked to the risk of developing ALS, but of course we cannot exclude the idea that other aspects of mitochondrial function not captured by the genetic measurements we have made, are determinants of ALS risk. Positioning of mitochondrial function as a disease modifier and not as a cause of ALS is important in the search for new therapeutic approaches. It is already known that high caloric diets can improve outcomes in ALS [39]. Targeted intervention aimed at boosting bioenergetic function is likely to be equally or even more effective and potentially synergistic.

## STAR methods

**Table:** Key resources table

Reagent or Resource	Source	Identifier
*Chemicals and Peptides*		
DMEM/Ham's F12 with Glutamax	Invitrogen	31331093
N2	Invitrogen	17502048
B27	Invitrogen	17504044
FGF-basic	Peprotech	100-18B
Fibronectin	Millipore	FC010
Accutase	Biolegend	423201
DAPT	Sigma	208255-80-5
Smoothened agonist	Millipore	364590-63-6
Forskolin	Cayman Chemical	66575-29-9
Retinoic acid	Sigma	4759-48-2
DNA2 inhibitor treatment C5	Generon	HY-128729
SYBR Green	Qiagen	204143
*Critical Commercial Assays*		
Neurite Outgrowth Staining Kit	ThermoFisher	A15001
Qiagen DNeasy kit	Qiagen	69504
*Experimental Models: Cell Lines*		
Fibroblasts obtained from ALS patients and controls described in Supplementary Table 15.	Primary cells	
*Software and Algorithms*		
R v4.2.1	https://cran.r-project.org/mirrors.html	
survival v3.5-0	https://cran.r-project.org/web/packages/survival/index.html	
survminer v0.4.9	https://cran.r-project.org/web/packages/survminer/index.html	
logistf v1.24.1	https://cran.r-project.org/web/packages/logistf/index.html	
PLINK V1.90	http://zzz.bwh.harvard.edu/plink/download.shtml	
ggplot2 v3.4.0	https://cran.r-project.org/web/packages/ggplot2/index.html	
TwoSampleMR v0.5.6	https://github.com/MRCIEU/TwoSampleMR	
VEP (Variant Effect Predictor)	https://github.com/Ensembl/ensembl-vep	
Harmony High-Content Imaging and Analysis Software	Perkin Elmer	

## Resource availability

### Lead Contact

Further information and requests for resources and reagents should be directed to and will be fulfilled by the Lead Contact, Johnathan Cooper-Knock (j.cooper-knock@sheffield.ac.uk).

### Materials availability

All unique/stable reagents generated in this study are available from the Lead Contact without restriction.

### Data and code availability

Whole genome sequencing (WGS) data for the Project MinE cohort is available through Project MinE (https://www.projectmine.com/research/data-sharing/). WGS and transcriptome data for the AnswerALS cohort is available from AnswerALS (https://www.answerals.org/). WGS for the PPMI cohort is available from the PPMI (https://www.ppmi-info.org/).

## Method details

Our approach and utilised datasets are summarised in Supplementary Fig. 6.

### Study cohort

The 5,594 sporadic unrelated ALS patients and 2,238 controls subject to WGS and included in this study as part of the Project MinE cohort were recruited at specialised neuromuscular centres in the UK, Belgium, Germany, Ireland, Italy, Spain, Turkey, the United States and the Netherlands [24]. Patients were diagnosed with possible, probable or definite ALS according to the 1994 El-Escorial criteria [40]. All controls were free of neuromuscular diseases and matched for age, sex and geographical location. This included sequencing of DNA obtained from whole blood, but also lymphoblastoid cells derived from 896 ALS patients and 400 controls, and CNS tissue from 133 ALS patients and 53 controls. Measurement of the ALS functional rating scale (ALSFRS-R) [30] was available for at least one timepoint in 1576 patients.

The AnswerALS cohort (https://www.answerals.org/) consisted of WGS in 843 ALS patients and 15 controls recruited from specialised neuromuscular centres in the United States. For a subset of 180 of these ALS patients, we obtained transcriptome data from iPSC-derived MN, and ALSFRS-R measurements.

The Parkinson's Progression Markers Initiative (PPMI) cohort (https://www.ppmi-info.org/) consists of WGS in 262 Parkinson's disease patients recruited from 50 international Neurology clinics. Clinical data is collected longitudinally including the unified Parkinson's disease rating scale (UPDRS) [41].

The study was approved by the South Sheffield Research Ethics Committee. Also, this study followed study protocols approved by Medical Ethical Committees for each of the participating institutions. Written informed consent was obtained from all participating individuals. All methods were performed in accordance with relevant national and international guidelines and regulations.

### Sample quality control

The Project MinE WGS dataset is recruited from a large number of different countries and healthcare settings. ALS survival is affected by access to healthcare and specific care delivered [42]. Kaplan-Meier curve analysis revealed heterogeneity in survival between Project MinE cohorts (Supplementary Fig. 1A) and atypically long survival [2] in Turkish and Portuguese cohorts. As a result Turkish and Portuguese cohorts, including 581 ALS patients, were excluded from our initial analysis.

### Mitochondrial haplotype

We tested for associations between mitochondrial haplotypes, defined by six mitochondrial SNPs. The derivation of these SNPs and haplotypes are described fully by Longchamps et al. [19]. Briefly, SNPs were identified by testing mitochondrial SNPs for independent effects on 41 mitochondrial related phenotypes within the UK Biobank cohort of 365,781 unrelated individuals. This identified 6 SNPs which were subsequently used to define haplotypes. Within the UK biobank cohort eight haplotypes were present with a MAF>0.05, and these haplotypes were robustly associated with non-cancer mortality, mtCN, and multiple mtCN associated traits including red blood cell characteristics, kidney function, and liver function. In our study, all ALS and control samples were assigned to a single haplotype. One haplotype was present in <2.5 % of ALS samples and therefore this haplotype was excluded from further analysis. The six mitochondrial SNPs used were MT73A_G, MT7028C_T, MT10238T_C, MT12612A_G, MT13617T_C, MT15257G_A (revised Cambridge reference sequence, reference allele first, alternate second). Haplogroups are displayed as ordered MT73_MT7028_MT10238_MT12612_MT13617_MT15257, with 2 as the reference allele, 0 as the alternate allele e.g. 2_2_2_2_2_2 would be the reference allele at every position whereas 0_2_2_2_2_2 would have the alternate allele for SNP MT73. The relative frequencies of all haplotypes are shown in Supplementary Fig. 1B. In subsequent analyses the most common haplogroup (2_2_2_2_2_2) was set as the reference and significance for haplogroup associations was generated by comparison to the reference group.

### Mitochondrial DNA copy number (mtCN)

Whole genome sequencing (WGS) has been established as the gold standard method for measuring mtCN [43] due to high depth (>2000X) coverage over the mitochondrial genome. MtCN was estimated using the ratio of reads aligning to mitochondrial DNA: the mtCN was calculated as double the number of reads aligning to a set of non-repetitive regions throughout the autosome. These non-repetitive regions were identified as control regions for the profiling of repeat expansions [44] and were transposed to build 37 for use with the Project MINE data using the UCSC liftover tool.

### White blood cell proportions

White blood cell (WBC) proportions including granulocytes, monocytes, natural killer (NK) cells, T-cells (CD4^+^ and CD8^+^) and B cells were estimated from methylation data in the Project MinE cohort as previously described [45]. Platelets counts were not available as they cannot be inferred from methylation data which may impact on the estimation of mtCN, because platelets contain mitochondrial DNA but no autosomal DNA [16].

### Statistical analysis including mitochondrial haplotypes and mtCN

All time-to-event analyses including survival and age of ALS onset were conducted using a Cox proportional hazards model. Analysis of the relationship between mitochondrial haplotype and mtCN utilised multivariable linear regression. Analysis of the relationship between MN gene expression or mtCN and ALSFRS-slope utilised multivariable linear regression. Analysis of the relationship between mitochondrial haplotype or mtCN and ALS status was conducted by multivariable logistic regression. All analyses included age, sex, site of disease onset, the first 20 principal components of genetic variation, and sequencing platform as covariates. Additional covariates are specified in the relevant sections. The sequencing platform was not available for the AnswerALS dataset.

We measured seven mitochondrial haplogroups which were considered as categorical variables; each individual haplotype was compared to the most frequent haplogroup 2_2_2_2_2_2 which was designated as the reference. We report the results of these analyses in our multivariable comparisons, but to assess the significance of haplotype overall we performed an ANOVA between regression models with and without the consideration of mitochondrial haplotypes. ANOVA enables comparison of the deviance between the model fits based on the log partial likelihood.

In survival analysis, left truncation bias [46] was corrected by considering only the time between patient sampling and time of death. Left truncation bias occurs when risk of death is measured over a time in which it could not have occurred; by definition a patient who had already died could not have been recruited into a study. The rationale is to exclude survival time that occurred before sampling, because the patient could not, by definition, have died during this period. Analysis of left truncated data can lead to false positive associations [46], however, left truncation bias correction can lead to under-powered analysis because of the loss of information. As a result, we report both results.

For multivariable linear and logistic regression analyses we applied the variance inflation factor to check for multicollinearity. White blood cell (WBC) proportions had a variance inflation factor >2 due to tightly correlated counts of T-cell subtypes. By removing NK cell and CD8^+^ T-cell counts (leaving CD4^+^ T-cells, granulocytes, monocytes and B cells) we returned a model where all covariates had a variance inflation factor <2. For all analyses of mtCN we therefore utilised CD4^+^ T-cells, granulocytes, monocytes and B cells, but not NK cell and CD8^+^ T-cell counts, as covariates.

### Transcriptome analysis

For AnswerALS data, gene expression profiling of iPSC-derived MNs and phenotype data were obtained for 180 ALS patients (https://www.answerals.org/). Gene expression was normalised for gene length and then sequencing depth to produce transcripts per kilobase million (TPM). We used multivariable linear regression to determine the relationship between gene expression and rate of change in the ALSFRS while including sequencing platform, sex, site of onset (spinal or bulbar), *C9ORF72* status, and the first 20 principal components of genetic variation as covariates. Significance testing was performed for all genes (n = 30,807) expressed in MNs as determined by mean TPM>1. In an unbiased analysis no gene was significantly associated after multiple testing correction but there was no evidence of inflation or deflation in the test statistics (Supplementary Fig. 2). To compare the distribution of p-values between genes linked to mitochondrial function and the background transcriptome we performed 10,000 permutations in which we constructed a gene set of the same size as the mitochondrial gene list; we then compared the median p-value for mitochondrial genes with the randomly chosen gene sets.

### Rare variant association testing

For analysis of WGS data from 5,594 sporadic ALS patients and 2,238 controls [24], variants within coding regions were determined to be rare if the minor allele frequency (MAF) within the Genome Aggregation Database (gnomAD) is < 1/100 control alleles [47]. In coding regions, we annotated variants using Variant Effect Predictor (VEP) [48]; Loss-of-function (LoF) variants were defined as nonsense mutations, high-effect splice-site mutations [49], or 5′UTR variants involving a gain/loss of a start/stop codon [50]*.* We applied a Cox proportional hazards model to determine the relationship between the number of rare LoF variants per patient and disease survival or censored survival time. Sequencing platform, sex, site of onset (spinal or bulbar), *C9ORF72* status, and the first 20 principal components of genetic variation were included as covariates. To test for a link between the number of rare LoF variants per patient and risk of ALS we used Firth logistic regression as previously described [51]. We excluded tests including <5 variants as these are likely to be underpowered. As with the transcriptome analysis, to compare the distribution of p-values between genes linked to mitochondrial function and the background exome we performed 10,000 permutations in which we constructed a gene set of the same size as the mitochondrial gene list; we then compared the median p-value for mitochondrial genes with the randomly chosen gene sets.

### Mendelian randomization (MR)

Ninety-three genomewide significant (p < 5E-8) autosomal SNPs were derived from the largest GWAS to-date [19] and utilised as instruments to measure genetic liability to increased mtCN. Identified SNPs within a 10 kb window were clumped for independence using a stringent cut-off of R^2^ ≤ 0.001 within a European reference panel; where SNPs were in linkage disequilibrium (LD) those with the lowest p-value were retained. The largest published ALS GWAS [4] was used to measure the effect of instrumental SNPs on risk of ALS. To measure the effect of instrumental SNPs on ALS survival we utilised the Project MinE dataset: SNPs linked to mtCN were encoded as 0,1,2 based on the number of effect alleles and Cox regression was used to measure the effect of allelic load on ALS survival after correcting for both WBC proportions and mitochondrial haplotype. The effects of SNPs on outcomes and exposures were harmonised in order to ensure that the beta values were signed with respect to the same alleles. Significance was calculated using an inverse variance weighted (IVW) estimate [52] which is well powered providing there is not an excess of invalid SNPs [53]. To increase confidence in the IVW results we performed a series of robust MR measures which are relatively robust to the presence of invalid SNPs, including the weighted median [53], weighted mode [54], and MR Egger [55]. MR analyses were performed using TwoSampleMR (v0.5.6).

### iNPC tissue culture, iNeuron differentiation and DNA2 inhibitor treatment

Induced Neuronal Progenitor Cells (iNPC's) were generated as previously described [56]. iNPC's were maintained in DMEM/Ham's F12 with Glutamax (Invitrogen) supplemented with N2, B27 (Invitrogen) and FGF-basic (Peprotech) on fibronectin (Millipore) coated cell culture dishes. Cells were routinely subcultured every 2–3 days using Accutase (Biolegend) to detach them. To achieve neuronal differentiation into iNeurons, iNPC's were plated into 6 well plates and cultured for 2 days in DMEM:F12 media with Glutamax (Invitrogen) supplemented with 1 % N2, 2 % B27 (Invitrogen), and 0.5 μM DAPT (Sigma). On day 3, DAPT was removed and media was supplemented with 0.5 μM smoothened agonist (SAG, Millipore), 2.5 μM Forskolin (Cayman Chemical) and 1 μM retinoic acid (Sigma) for 16 days. For treatments with DNA2 inhibitor treatment C5 (Generon), cells were treated for 72 h with 75 μM before being assayed.

### Live cell imaging assays of iNeurons

Cell viability was measured using a viability indicator from Neurite Outgrowth Staining Kit (ThermoFisher) as per manufacturer's instructions. For mitochondrial membrane potential (MMP) quantification, iNeurons were treated with 80 nM TMRM (Sigma) and 100 nM Mitotracker Deep Red (ThermoFisher) for 1 h prior to imaging. Cells were imaged using an Opera Phenix high content imaging system, with analysis performed using a custom protocol on Harmony software (PerkinElmer). Nuclei counts after DNA2 inhibitor treatment with C5 (Generon) were normalised to baseline counts per cell line.

### DNA extraction and mtDNA quantification for iNeurons

Total DNA was extracted using the Qiagen DNeasy kit using manufacturer's instructions. mtDNA was quantified by presence of the mitochondrial complex I gene ND4 [57]. PCR reactions were performed as 20 μl reactions. 100 ng of DNA was loaded per well with final primer concentrations of 300 nM. Reactions were performed in triplicate with SYBR Green (Qiagen) on a C1000 Touch thermocycler using the CFX96 Real-Time System (Biorad). Samples were initially denatured for 10 min at 95°, followed by 40 cycles of: 95° for 15 s, 60° for 1 min (followed by fluorescence reading), 72° for 1 min. Melt curves were recorded following the last cycle. Each MtCN measurement was performed for a pair of case and control cell lines. In downstream analyses each mtCN value was normalised to the value for the control sample within each pair. ND4 primer sequences: ND4-F: 5′-CCATTCTCCTCCTATCCCTCAAC-3′;

ND4-R: 5′-CACAATCTGATGTTTTGGTTAAACTATATTT-3′. U1 primer sequences: U1–F: 5′-CCATGATCACGAAGGTGGTT-3’; U1-R: 5′-ATGCAGTCGAGTTTCCCACA-3’.

## CRediT authorship contribution statement

**Calum Harvey:** Writing – review & editing, Writing – original draft, Visualization, Validation, Methodology, Investigation, Formal analysis, Data curation, Conceptualization. **Marcel Weinreich:** Writing – review & editing, Writing – original draft, Visualization, Validation, Software, Methodology, Investigation, Formal analysis, Data curation. **James A.K. Lee:** Writing – review & editing, Writing – original draft, Visualization, Validation, Methodology, Investigation, Formal analysis, Data curation. **Allan C. Shaw:** Writing – review & editing, Methodology, Data curation. **Laura Ferraiuolo:** Writing – review & editing, Investigation, Funding acquisition, Formal analysis, Data curation. **Heather Mortiboys:** Writing – review & editing, Writing – original draft, Visualization, Validation, Project administration, Methodology, Investigation, Funding acquisition, Formal analysis, Data curation. **Sai Zhang:** Writing – review & editing, Writing – original draft, Visualization, Validation, Methodology, Investigation, Formal analysis. **Paul J. Hop:** Writing – review & editing, Formal analysis, Data curation. **Ramona A.J. Zwamborn:** Writing – review & editing, Formal analysis, Data curation. **Kristel van Eijk:** Writing – review & editing, Formal analysis, Data curation. **Thomas H. Julian:** Writing – review & editing, Writing – original draft, Methodology, Investigation. **Tobias Moll:** Writing – review & editing, Writing – original draft, Visualization, Validation, Project administration, Methodology, Investigation, Formal analysis, Data curation. **Alfredo Iacoangeli:** Writing – review & editing, Formal analysis, Data curation. **Ahmad Al Khleifat:** Writing – review & editing, Formal analysis, Data curation. **John P. Quinn:** Writing – review & editing, Formal analysis, Data curation. **Abigail L. Pfaff:** Writing – review & editing, Methodology, Formal analysis, Data curation. **Sulev Kõks:** Writing – review & editing, Supervision, Software, Funding acquisition, Formal analysis, Data curation. **Joanna Poulton:** Writing – review & editing, Methodology, Formal analysis. **Stephanie L. Battle:** Writing – review & editing, Software, Formal analysis, Data curation. **Dan E. Arking:** Writing – review & editing, Supervision, Software, Methodology, Funding acquisition, Formal analysis, Data curation. **Michael P. Snyder:** Writing – review & editing, Writing – original draft, Supervision, Software, Project administration, Funding acquisition, Formal analysis. **Jan H. Veldink:** Writing – review & editing, Supervision, Project administration, Funding acquisition, Formal analysis. **Kevin P. Kenna:** Writing – review & editing, Writing – original draft, Visualization, Validation, Supervision, Funding acquisition, Formal analysis, Data curation. **Pamela J. Shaw:** Writing – review & editing, Writing – original draft, Supervision, Project administration, Funding acquisition, Formal analysis. **Johnathan Cooper-Knock:** Writing – review & editing, Writing – original draft, Visualization, Validation, Supervision, Software, Resources, Project administration, Methodology, Investigation, Funding acquisition, Formal analysis, Data curation, Conceptualization.

## Declaration of competing interest

The authors declare the following financial interests/personal relationships which may be considered as potential competing interests:Michael P Snyder reports a relationship with Personalis Inc that includes: board membership. Michael P Snyder reports a relationship with Q Bio that includes: board membership. Michael P Snyder reports a relationship with SensOmics that includes: board membership. Michael P Snyder reports a relationship with January that includes: board membership. Michael P Snyder reports a relationship with Protos that includes: board membership. Michael P Snyder reports a relationship with Mirvie that includes: board membership. Michael P Snyder reports a relationship with NiMo that includes: board membership. Michael P Snyder reports a relationship with Onza that includes: board membership. Michael P Snyder reports a relationship with Oralome that includes: board membership. Michael P Snyder reports a relationship with Danaher that includes: board membership. Michael P Snyder reports a relationship with GenapSys Inc that includes: board membership. Michael P Snyder reports a relationship with Jupiter that includes: board membership.
