# Supplementary figures and images for: Rare and common genetic determinants of mitochondrial function determine severity but not risk of amyotrophic lateral sclerosis
Source: Heliyon. 2024 Jan 24;10(3):e24975. doi: 10.1016/j.heliyon.2024.e24975 (PMC10839612; doi:10.1016/j.heliyon.2024.e24975)

Supplementary Figure 1

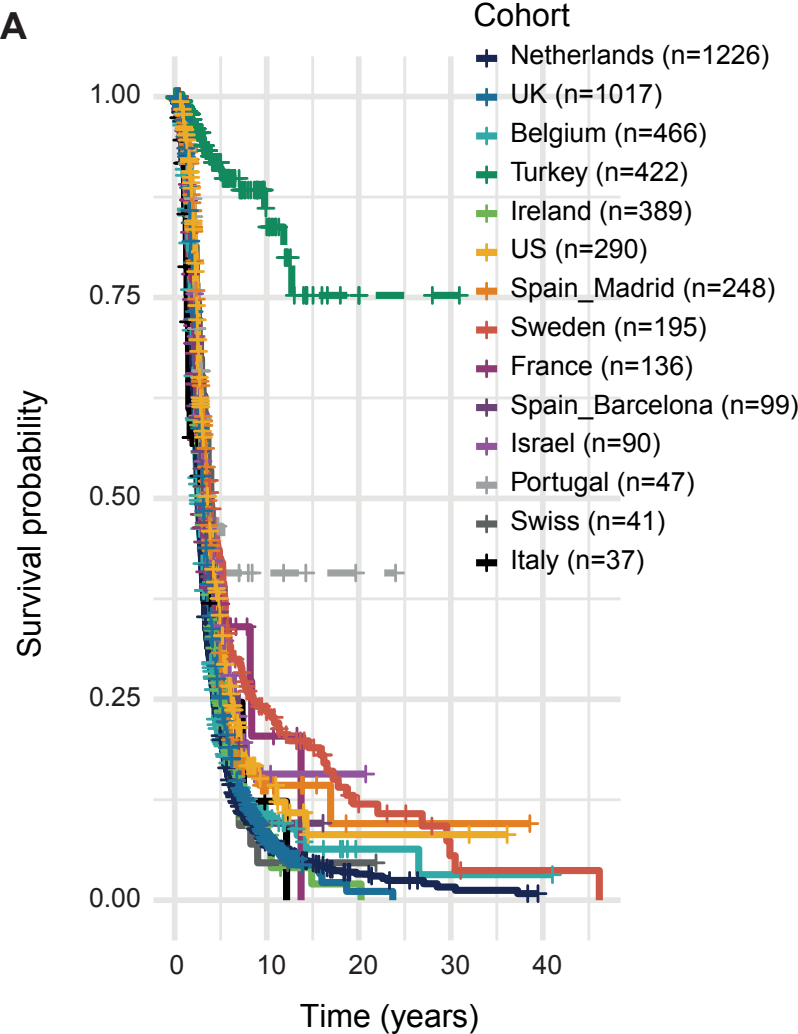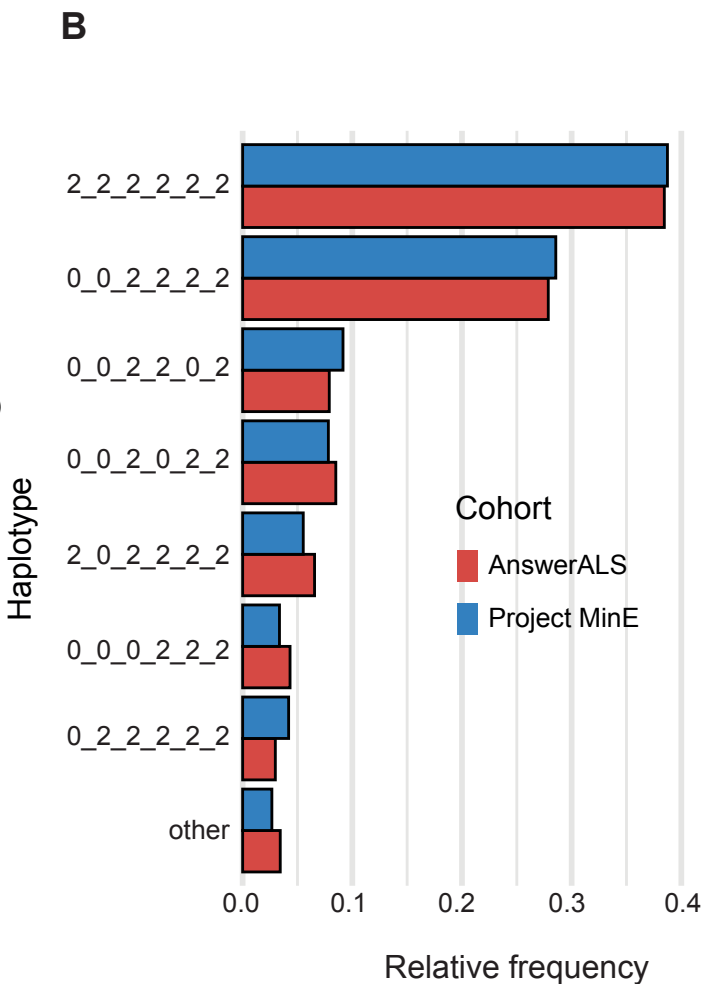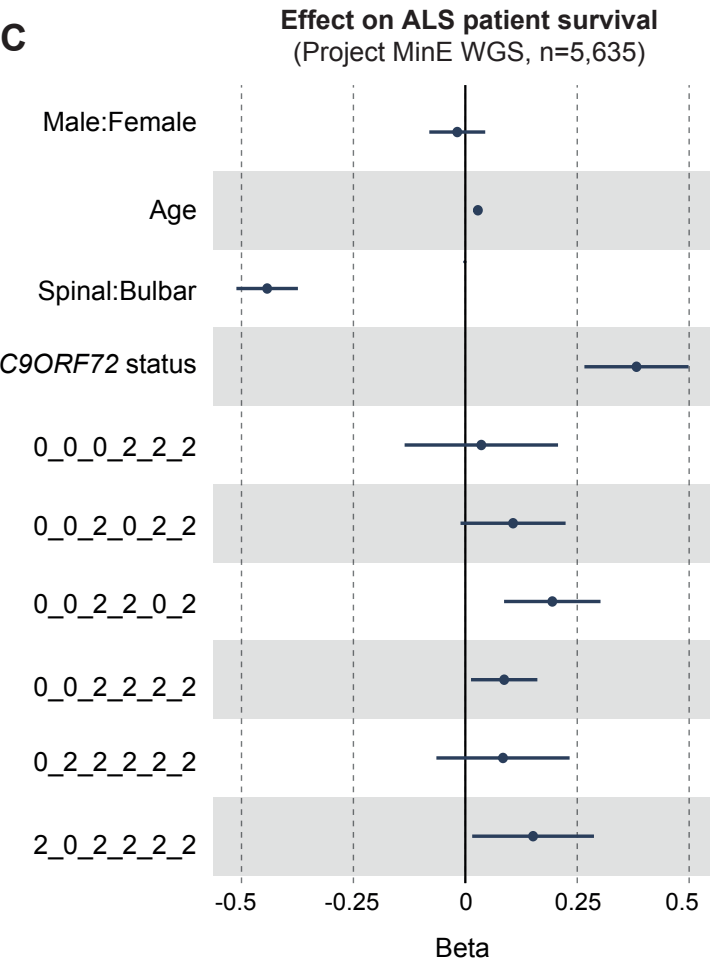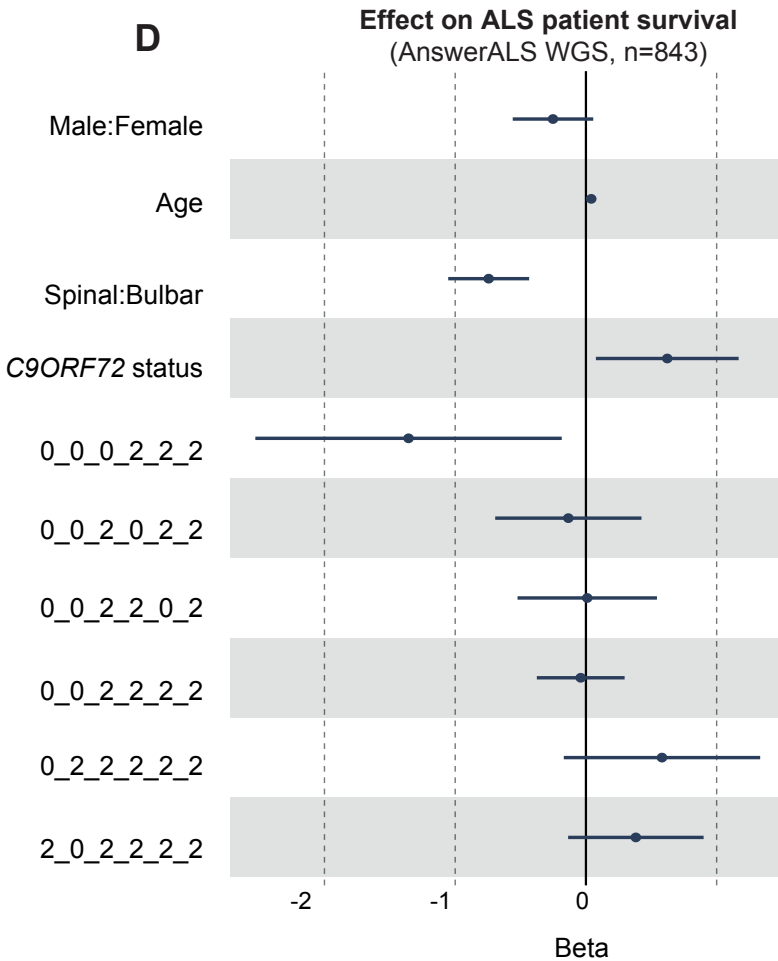

Supplement: Multimedia component 1 [file mmc1.pdf]

Supplementary Figure 2

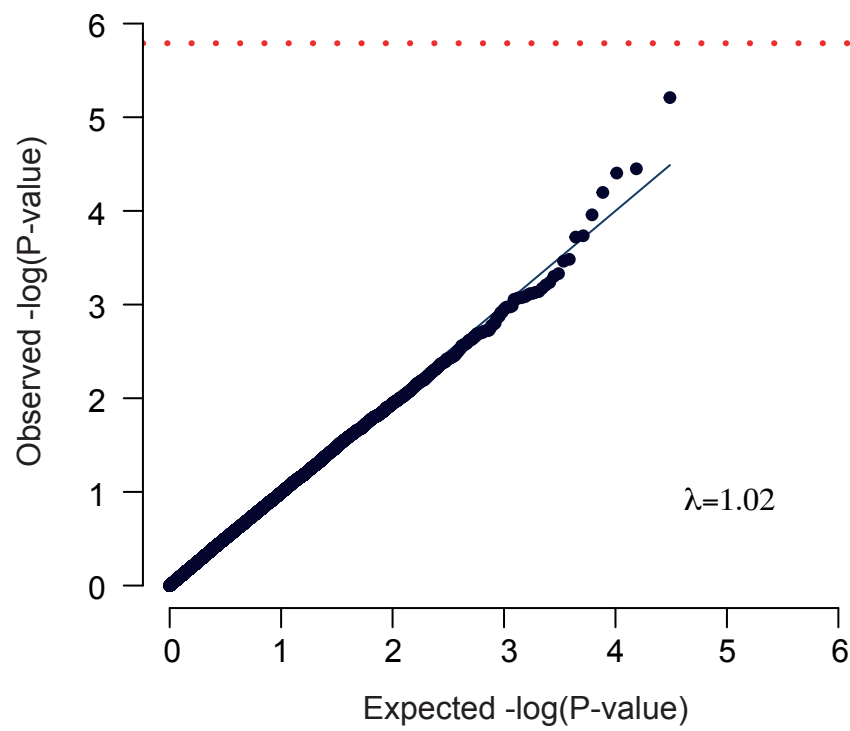

Supplement: Multimedia component 2 [file mmc2.pdf]

Supplementary Figure 4

Key

A

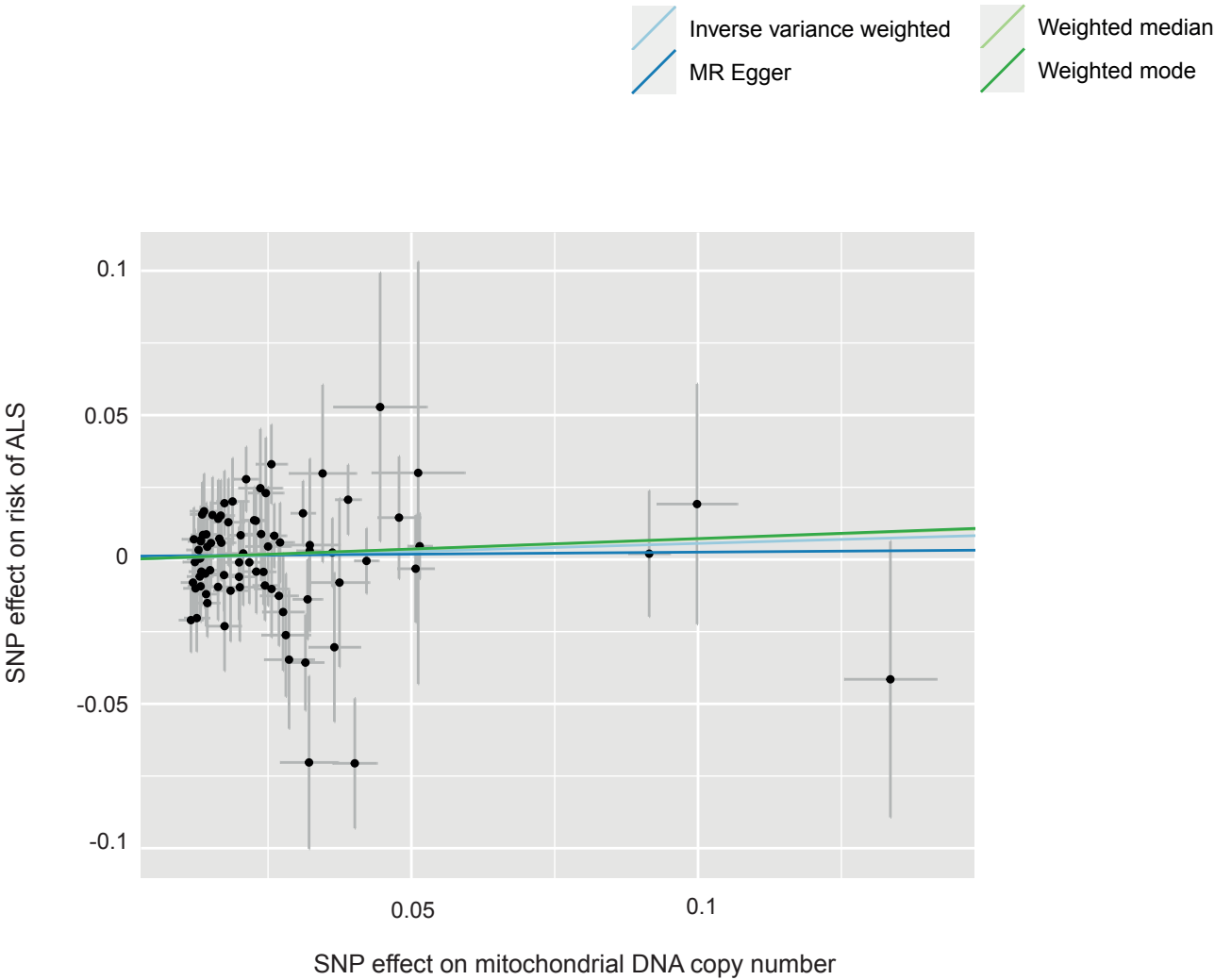

B

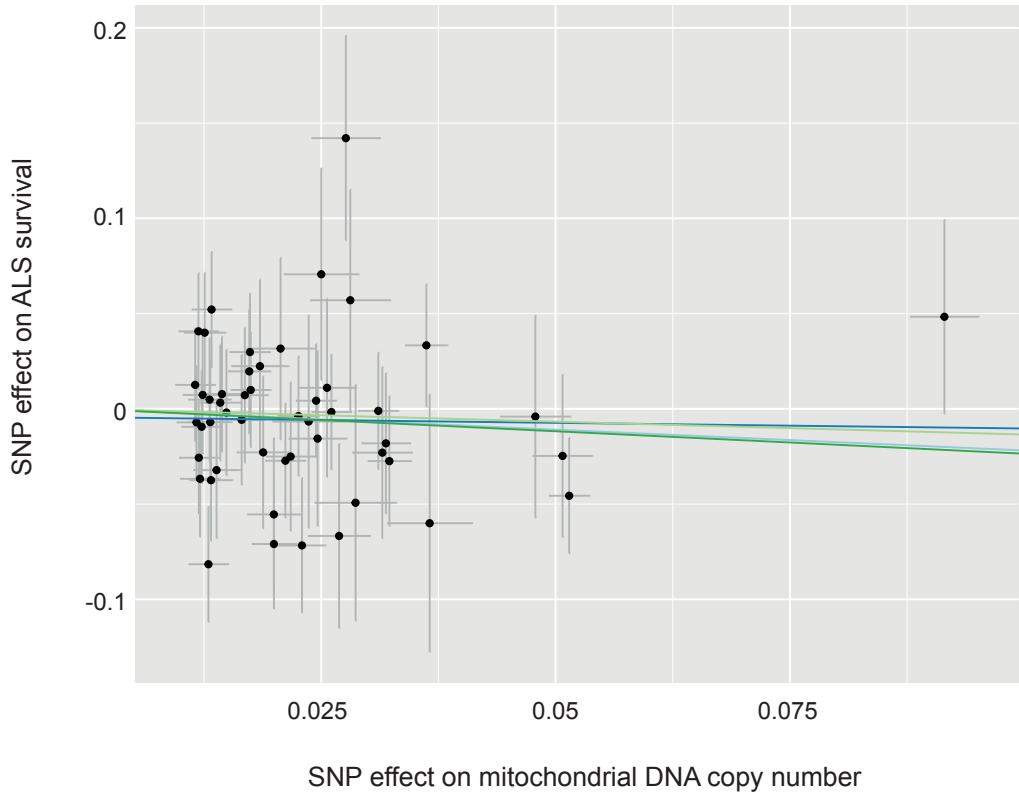

Supplement: Multimedia component 4 [file mmc4.pdf]

# Supplementary Figure 5

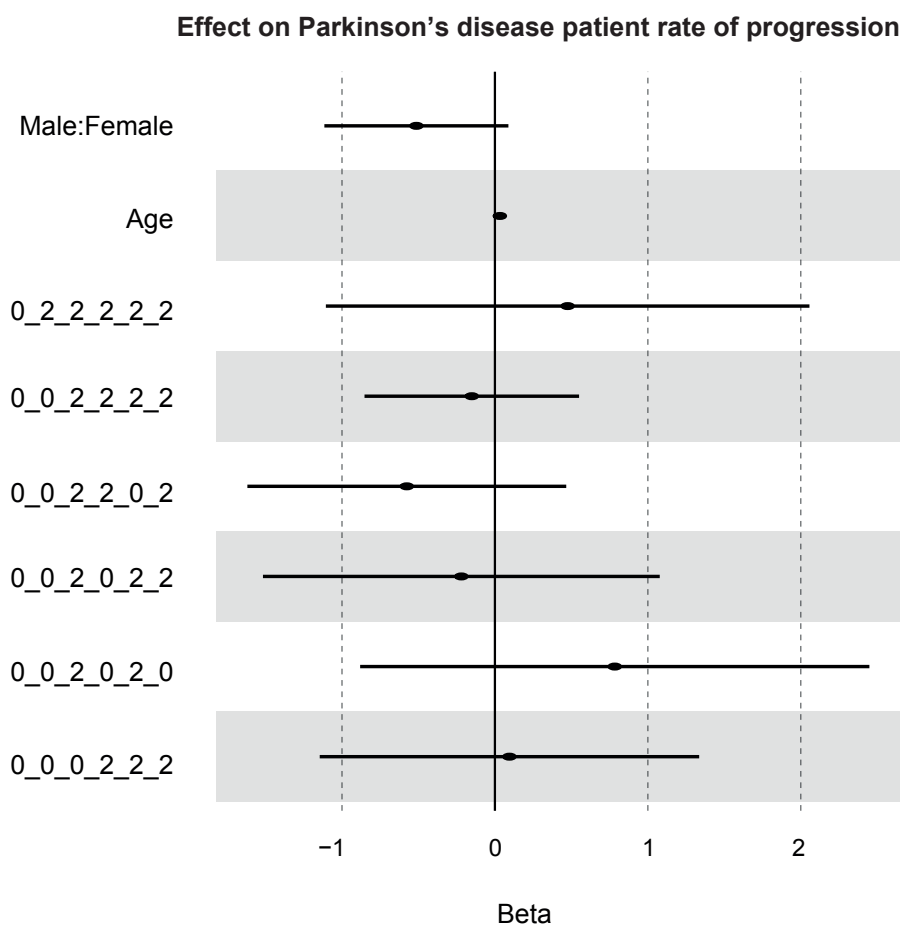

Supplement: Multimedia component 5 [file mmc5.pdf]
