# Supplementary material for: Rare and common genetic determinants of mitochondrial function determine severity but not risk of amyotrophic lateral sclerosis
Source: Heliyon. 2024 Jan 24;10(3):e24975. doi: 10.1016/j.heliyon.2024.e24975 (PMC10839612; doi:10.1016/j.heliyon.2024.e24975)

Supplementary Figure 3

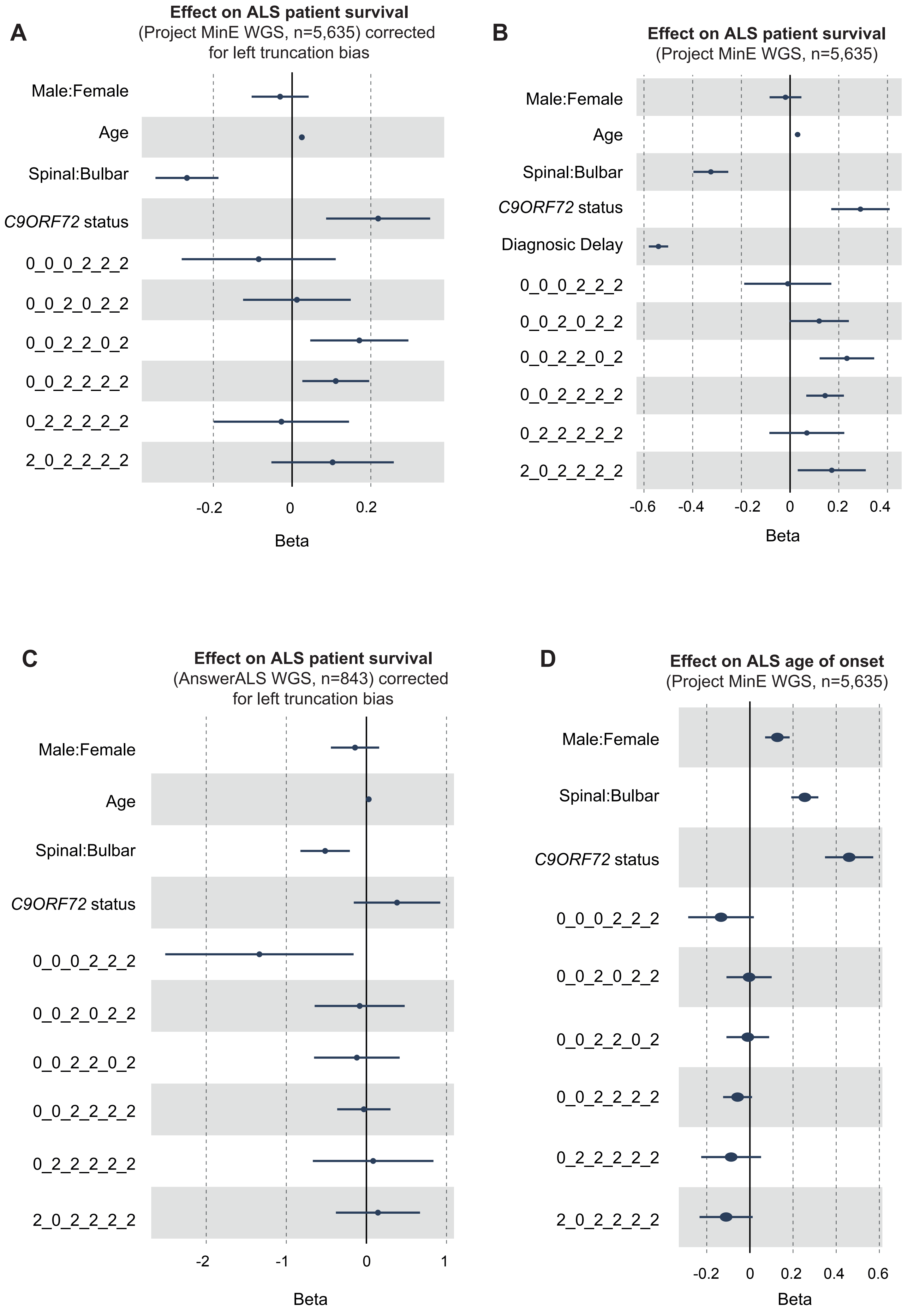

Supplement: Multimedia component 3 [file mmc3.pdf]
