# Supplementary material for: Rare and common genetic determinants of mitochondrial function determine severity but not risk of amyotrophic lateral sclerosis
Source: Heliyon. 2024 Jan 24;10(3):e24975. doi: 10.1016/j.heliyon.2024.e24975 (PMC10839612; doi:10.1016/j.heliyon.2024.e24975)
Supplement: Multimedia component 6 [file mmc6.pdf]

# Supplementary Figure 6

## Methods

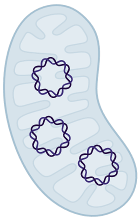

Mitochondrial function is inferred from mitochondrial genetic haplotypes

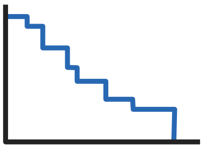

Inferred mitochondrial function was tested for an effect on ALS risk and survival

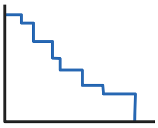

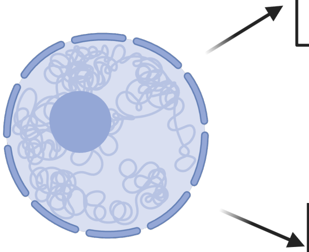

Loss-of-function (LoF) mutations within nuclear encoded mitochondrial genes were tested for an effect on ALS survival

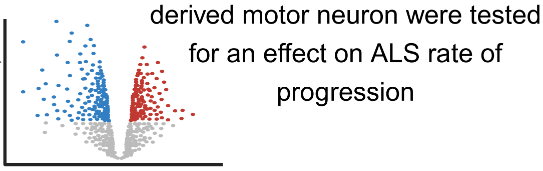

Expression of nuclear encoded mitochondrial genes within iPSC-derived motor neuron were tested for an effect on ALS rate of progression

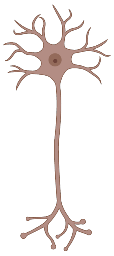

The effect of LoF mutations within *DNA2*, a nuclear encoded mitochondrial gene linked to ALS survival, were phenocopied in patient-derived iNeurons to determine the effect on cell survival and mitochondrial function

## Datasets

(1) WGS from Project MinE (n=5,954 ALS patients and n=2,238 controls).
(2) WGS from AnswerALS (n=843 ALS patients).

(1) WGS from Project MinE (n=5,954 ALS patients and n=2,238 controls).
(2) RNAseq from AnswerALS patient-derived motor neurons (n=180).

iNeurons derived from n=3 C9ORF72 ALS patients and n=3 age and sex matched controls.
