# Supplementary material for: Rare and common genetic determinants of mitochondrial function determine severity but not risk of amyotrophic lateral sclerosis
Source: Heliyon. 2024 Jan 24;10(3):e24975. doi: 10.1016/j.heliyon.2024.e24975 (PMC10839612; doi:10.1016/j.heliyon.2024.e24975)
Supplement: Multimedia component 8 [file mmc8.docx]

**Supplementary Figure 1: Survival characteristics and analysis of the effect of individual mitochondrial haplotypes on ALS patient survival.** (A) Kaplan-Meier curve revealed significant heterogeneity between sub-cohorts. As a result of atypical characteristics the Turkish and Portuguese cohorts were excluded from the initial analysis. Censored survival times are represented by a cross. (B) Relative frequency for each of the studied mitochondrial haplotypes in both the Project MinE and AnswerALS cohorts of ALS patients. 2_2_2_2_2_2 was the most frequent and was taken as the reference haplotype. Only the six haplotypes present in >2.5% of the study cohort were included in further analyses to avoid underpowered tests. Mitochondrial haplotypes were determined by six mitochondrial SNPs: MT73A_G, MT7028C_T, MT10238T_C, MT7028C_T, MT12612A_G, MT13617T_C, MT15257G_A. Haplogroups are displayed as ordered MT73_MT7028_MT10238_MT12612_MT13617_MT15257, with 2 as the reference allele, 0 as the alternate allele. (C) Forest plot for the effect of mitochondrial haplotypes, sex, age, site of onset and *C9ORF72* status on ALS survival in samples from the Project MinE cohort (n=5,635) tested by Cox regression. (D) Forest plot for the effect of mitochondrial haplotypes, sex, age, site of onset and *C9ORF72* status on ALS survival in samples from the AnswerALS cohort (n=843) tested by Cox regression.

**Supplementary Figure 2: QQ-plot for expected and observed P-values in multivariable linear regression analysis of the effect of gene expression within iPSC-derived motor neurons on rate of ALS progression.** The test statistics show no evidence of inflation (λ=1.02) but no individual gene is significant after Bonferroni multiple testing (indicated by red line). 30,807 genes were tested. ALS progression was measure by the rate of change in the ALS functional rating score (ALSFRS).

**Supplementary Figure 3: Analysis of the effect of mitochondrial haplotype on ALS patient survival and age of onset** (A) Forest plot for Cox regression penalised to correct for left truncation bias, for the effect of mitochondrial haplotype, sex, age, site of onset and disease status on ALS survival in samples from the Project MinE cohort. (B) Forest plot for Cox regression for the effect of mitochondrial haplotype, sex, age, site of onset, *C9ORF72* status, and diagnostic delay on ALS survival in samples from the Project MinE cohort. (C)  Forest plot for Cox regression penalised to correct for left truncation bias, for the effect of mitochondrial haplotype, sex, age, site of onset and disease status on ALS survival in samples from the AnswerALS cohort. (D) Forest plot for Cox regression for the effect of mitochondrial haplotype, sex, site of onset, *C9ORF72* status, on ALS age of onset in samples from the Project MinE cohort.

**Supplementary Figure 4:**  **Mendelian  randomization (MR) analysis of the effect of genetic liability to increased mtCN on ALS risk and severity using autosomal SNPs.** Scatter plots demonstrating the correlation between genetic liability to increased mtCN on genetic liability to ALS (A) and genetic liability to reduced ALS patient survival (B). Points indicate effect size (beta) and standard errors for each SNP-outcome relationship.

**Supplementary Figure 5: Mitochondrial haplotype is not linked to Parkinson’s disease (PD) rate of progression.** (A) Forest plot for Cox regression for the effect of mitochondrial haplotype, sex, and age on PD progression as quantified by rate of change in the UDPRS score measured at 5 years after symptom onset in 262 PD patients.

**Supplementary Figure 6: Workflow including analyses and utilised datasets**

**Supplementary Table 1: Effect sizes and test statistics for multivariable linear regression including the effect of mitochondrial haplotype on mitochondrial copy number.** This analysis was carried out in blood samples from the Project MinE cohort including 3,549 sporadic ALS patients and 1,529 controls.

**Supplementary Table 2: Effect sizes and test statistics for Cox regression including the effect of mitochondrial haplotype on ALS survival.** This analysis was carried out in samples from the Project MinE cohort including 5,635 sporadic ALS patients.

**Supplementary Table 3: Effect sizes and test statistics for Cox regression penalised to correct for left truncation bias including the effect of mitochondrial haplotype on ALS survival.** This analysis was carried out in samples from the Project MinE cohort including 5,635 sporadic ALS patients.

**Supplementary Table 4: Effect sizes and test statistics for Cox regression including the effect of mitochondrial haplotype and diagnostic delay on ALS survival.** This analysis was carried out in samples from the Project MinE cohort including 5,635 sporadic ALS patients.

**Supplementary Table 5: Effect sizes and test statistics for Cox regression including the effect of mitochondrial haplotype on ALS survival.** This analysis was carried out in the AnswerALS cohort including 843 ALS patients.

**Supplementary Table 6: Effect sizes and test statistics for Cox regression penalised to correct for left truncation bias including the effect of mitochondrial haplotype on ALS survival.** This analysis was carried out in the AnswerALS cohort including 843 ALS patients.

**Supplementary Table 7: Effect sizes and test statistics for multivariable logistic regression including the correlation between mitochondrial copy number and ALS.** This analysis was carried out in blood samples from the Project MinE cohort including 3,549 sporadic ALS patients and 1,529 controls.

**Supplementary Table 8: Effect sizes and test statistics for Cox regression including the correlation between mitochondrial copy number and ALS survival.** This analysis was carried out in blood samples from the Project MinE cohort including 3,549 sporadic ALS patients.

**Supplementary Table 9: Effect sizes and test statistics for Cox regression including the correlation between mitochondrial copy number and ALS age of onset.** This analysis was carried out in blood samples from the Project MinE cohort including 3,549 sporadic ALS patients.

**Supplementary Table 10: Effect sizes and test statistics for Cox regression including the correlation between mitochondrial copy number and age at sampling in controls.** This analysis was carried out in blood samples from the Project MinE cohort including 1,529 controls.

**Supplementary Table 11: Effect sizes and test statistics for Cox regression including the effect of mitochondrial haplotype on ALS age of onset.** This analysis was carried out in samples from the Project MinE cohort including 5,635 sporadic ALS patients.

**Supplementary Table 12:  Mendelian randomisation measures for the effect of genetic liability to increased mitochondrial DNA copy number measured using autosomal SNPs, on genetic liability to ALS and genetic liability to reduced ALS survival.**

**Supplementary Table 13:  Effect sizes and test statistics for multivariable linear regression including the effect of mitochondrial haplotype on rate of Parkinson’s disease (PD) progression.** This analysis was carried out in blood samples from the PPMI cohort including 262 sporadic PD patients.

**Supplementary Table 14: Effect sizes and test statistics for Cox regression including the effect of burden of rare loss of function genetic variants within nuclear encoded genes associated with mitochondrial function on ALS survival.** This analysis was carried out in samples from the Project MinE cohort including 5,635 sporadic ALS patients.

**Supplementary Table 15: Effect sizes and test statistics for multivariable linear regression including the effect of expression by iPSC-derived motor neurons of nuclear encoded genes associated with mitochondrial function on rate of ALS progression.** This analysis was carried out in the AnswerALS cohort including 180 ALS patients.

**Supplementary Table 16: ALS patients and controls who donated fibroblasts used to generate iNeurons.**

**Supplementary Table 17: Project MinE ALS Sequencing Consortium**
